# Residual effects of zopiclone 7.5 mg on highway driving performance in insomnia patients and healthy controls: a placebo controlled crossover study

**DOI:** 10.1007/s00213-014-3447-z

**Published:** 2014-01-24

**Authors:** T. R. M. Leufkens, J. G. Ramaekers, A. W. de Weerd, W. J. Riedel, A. Vermeeren

**Affiliations:** 1 Division Information and Cognition, Department of Brain, Body and Behavior, Philips Group Innovation–Research, High Tech Campus 34, 5656 AE Eindhoven, The Netherlands; 2 Experimental Psychopharmacology Unit, Department of Neuropsychology and Psychopharmacology, Faculty of Psychology and Neuroscience, Maastricht University, Maastricht, The Netherlands; 3 Department of Clinical Neurophysiology and Sleep Centre SEIN, Zwolle, The Netherlands

**Keywords:** Zopiclone, Hypnotics, Residual effects, Insomnia, On-the-road driving

## Abstract

**Rationale:**

Residual effects of hypnotics on driving performance have been mainly determined in studies using a standardized driving test with healthy good sleepers. Responses to effects may differ, however, between insomniacs and healthy volunteers due to the underlying sleep disorder. In addition, a majority of insomniacs uses hypnotics chronically resulting in the development of tolerance to impairing effects. Impaired driving performance in healthy volunteers may then be an overestimation of the actual effects in insomniacs.

**Objectives:**

The present study aims to compare the residual effects of zopiclone 7.5 mg on on-the-road driving performance of 16 middle-aged insomniacs chronically using hypnotics (chronic users), 16 middle-aged insomniacs not or infrequently using hypnotics (infrequent users), and 16 healthy, age matched, good sleepers (controls).

**Methods:**

The study was conducted according to a 3 × 2 double-blind, placebo controlled crossover design, with three groups and two treatment conditions. Treatments were single oral doses of zopiclone 7.5 mg and placebo administered at bedtime (2330 hours). Between 10 and 11 h after administration subjects performed a standardized highway driving test.

**Results:**

Zopiclone 7.5 mg significantly impaired on-the-road driving performance in both insomnia groups and healthy controls. The magnitude of impairment was significantly less in the chronic users group as compared with the controls.

**Conclusions:**

The smaller magnitude of effects suggests that investigating residual effects of hypnotics in healthy volunteers may yield a minor overestimation of the actual effects in insomnia patients.

## Introduction

Residual daytime sedation is one of the main problems associated with hypnotic drug use. Experimental studies have demonstrated that the sedative actions of hypnotics impair psychomotor and cognitive functioning the morning after evening administration (Vermeeren 2004). The related reduced alertness and slowed reactions are a particular problem for individuals who have to drive a car the morning following an evening dose. Epidemiological studies have shown that use of benzodiazepines, as well as zopiclone, is associated with an increased risk of car accidents (Hemmelgarn et al. 1997; Barbone et al. 1998; Neutel 1998; Glass et al. 2005).

The severity and duration of residual effects on actual driving performance of hypnotics have been determined in experimental studies using a standardized driving test (Vermeeren 2004). Most of those studies have been conducted in healthy, young volunteers rather than in the target population, i.e., patients suffering from insomnia. Responses to the residual effects of hypnotics, however, may differ between insomnia patients and healthy good sleepers due to the underlying sleep disorder. In insomnia patients, hypnotics are expected to improve sleep and, as a consequence, they are expected to improve daytime performance as well. This improvement is supposed to attenuate or even compensate for the impairing effects of hypnotics. Few studies have assessed residual effects of hypnotics on on-the-road or simulated driving in insomnia patients (Vermeeren 2004). They show however that driving is also impaired in insomniacs. For example, residual effects in women complaining of insomnia have been found 10 h after administration of zopiclone 7.5 mg (Volkerts et al. 1984) and lormetazepam 2 mg (Brookhuis et al. 1994) in on-the-road driving. In addition, a study examining the effects of zopiclone 7.5 mg, zolpidem 10 mg, and lormetazepam 1 mg on simulated driving in insomniac patients showed that zopiclone and lormetazepam, but not zolpidem, impaired driving between 9 and 11 h after administration (Staner et al. 2005).

A second reason why responses to the residual effects of hypnotics may differ between insomnia patients and healthy good sleepers is that the majority of insomnia patients use hypnotics for prolonged periods (Curran et al. 2003). This may result in the development of tolerance to the impairing effects. Impaired driving performance found in healthy medication-naïve volunteers may then be an overestimation of the actual effects in insomnia patients.

Finally, most users of hypnotics are older (Drake et al. 2003; Glass et al. 2005). On the one hand, age is associated with changes in pharmacokinetics and pharmacodynamics which may increase patients’ vulnerability to impairing effects of hypnotics (Glass et al. 2005; Bocca et al. 2011). In that case, the effects found in studies with healthy young volunteers may be an underestimation of the effects in the target population of the drugs. On the other hand, driving experience increases with age, which may have a protective effect. For example, results from an on-the-road driving study in 18 healthy, older drivers (ages ranging between 56 to 73 years) showed that the effects of a hypnotic did not increase with age (Leufkens and Vermeeren 2009). If anything, the effects in older drivers seemed to be less severe than in younger drivers, but this needs to be replicated.

Although there already exists much insight about the residual effects of hypnotics on driving performance, a study directly comparing the effects between insomnia patients, chronic users of hypnotics, and healthy good sleepers has never been conducted. Recently, an observational study explored driving performance between pharmacologically treated and untreated older insomnia patients and healthy, age-matched, good sleepers (Leufkens et al. 2014; Ramaekers 2011). Results showed that performance was not significantly different between insomnia patients and healthy controls. In addition, there were no significant differences between insomnia patients who chronically used hypnotics and patients who used hypnotics infrequently. A limitation of that study was, however, that the chronic users group used a variety of hypnotic drugs most of which were not expected to produce residual sedation at all. In addition, variability in dose and half-life may have added to the absence of any performance impairment. In order to determine whether residual effects of hypnotics on driving performance are similar or reduced in insomnia patients as compared to healthy volunteers, studies need to be conducted with hypnotics that have been shown to produce residual impairment in healthy volunteer studies, such as zopiclone (Vermeeren et al. 1998, 2002a; Leufkens et al. 2009; Leufkens and Vermeeren 2009). A recent analysis of pooled data from four studies showed that the average effect of zopiclone 7.5 mg is equivalent to that found for alcohol in the same test when blood alcohol concentrations range between 0.05 and 0.08 % (Leufkens and Vermeeren 2014).

Therefore, the present study aims to compare the residual effects of the frequently prescribed hypnotic zopiclone 7.5 mg on driving performance of 16 older insomnia patients who chronically use hypnotics, 16 older insomnia patients who do not or infrequently use hypnotics, and 16 healthy, age-matched, good sleepers.

## Methods

### Subjects

All subjects in the present study participated in a previous study by Leufkens and Ramaekers (Leufkens et al. 2014; Ramaekers 2011). They were asked upon completion of the former study to continue their participation in the present study. In the previous study, insomnia patients, in the age range of 52 to 73 years, were initially recruited through a network of local general practitioners in the region of Maastricht, The Netherlands (Regionaal Netwerk Huisartsen). Possible candidates were selected from a computerized database of the Center for Data and Information Management of Maastricht University (MEMIC). This recruitment procedure was subsequently backed up by advertisement in local newspapers. Healthy controls were recruited by advertisements in local newspapers.

Three groups of 16 subjects, ranging from 52 to 71 years of age, participated in the present study. Groups were 16 individuals with insomnia who chronically used hypnotics (“chronic users”: seven females and nine males), 16 individuals with insomnia who did not or infrequently used hypnotics (“infrequent users”: eight females and eight males), and 16 self-defined good sleepers, matched for age and driving experience (“controls”: seven females and nine males). Their mean (±SD) ages were 62.6 (4.5) for the chronic users, 62.3 (6.2) for the infrequent users, and 62.9 (4.3) for the controls.

Insomnia patients had to meet the inclusion criteria for primary insomnia according to DSM-IV (Association AP 1994): (1) subjective complaints of insomnia, defined as difficulties initiating sleep (sleep latency >30 min) and/or maintaining sleep (awakenings >30 min); (2) duration of more than 1 month; (3) the sleep disturbance causes clinically significant distress or impairment; (4) insomnia does not occur exclusively during the course of a mental disorder; and (5) insomnia is not due to another medical or sleep disorder or effects of medication or drug abuse.

Sleep complaints were measured using Dutch versions of the Pittsburgh Sleep Quality Index (Buysse et al. 1989), the Sleep Wake Experience List (van Diest et al. 1989), and the general version of the Groningen Subjective Quality of Sleep questionnaire (Mulder-Hajonides van der Meulen 1981). Additionally, a daily journal and the specific version of the Groningen Subjective Quality of Sleep questionnaire (GSQS-spec) (Mulder-Hajonides van der Meulen 1981) were completed upon arising each morning for 2 weeks providing subjective estimates of sleep quality.

Insomnia patients were assigned to the “chronic users” group when they used a benzodiazepine, zopiclone, or zolpidem as sleeping medication for at least four nights per week during the previous 3 months or more. Patients not using hypnotics or using hypnotics less than or equal to 3 days per week were assigned to the “infrequent users” group. Self-defined good sleepers did not meet any of the criteria for insomnia and did not use any hypnotics. Table 1 presents an overview of the mean frequencies and durations of hypnotic use per group, as well as the individual hypnotics and doses used.

**Table 1:** Demographics, sleep diary data, and overview of hypnotic use for the insomnia groups (means and SD are displayed)

	Chronic users *n* = 16	Infrequent users *n* = 16	Controls *n* = 16
Age (years)	62.6 (4.5)	62.3 (6.2)	62.9 (4.3)
Gender (*n*)	7 females, 9 males	8 females, 8 males	7 females, 9 males
Pittsburgh Sleep Quality Index	11.9 (3.7)	12.4 (2.2)	2.5 (1.5)
Sleep Onset Latency (min)	43.6 (34.4)	60.8 (64.3)	14.0 (8.9)
Total Sleep Time (min)	381 (103)	306 (64)	448 (26)
Sleep Efficiency (%)	73.4 (14.1)	65.6 (14.2)	90.7 (7.3)
Number of users of hypnotics (%)	16 (100 %)	10 (62.5 %)	0 (0 %)
Frequency of hypnotic use (nights per week)	6.6 (1.0)	1.1 (0.8)	0
Duration of hypnotic use (years)	7.1 (5.0)	7.8 (8.1)	0
Hypnotics and doses used (n)			
Flurazepam 15 mg	1	0	–
Lorazepam 1 mg	0	1	–
Lormetazepam 0.5 mg	1	0	–
Lormetazepam 2 mg	1	0	–
Midazolam 7.5 mg	3	0	–
Nitrazepam 5 mg	1	1	–
Oxazepam 10 mg	1	0	–
Oxazepam 20 mg	1	0	–
Oxazepam 50 mg	1	0	–
Temazepam 10 mg	2	4	–
Temazepam 20 mg	1	1	–
Zopiclone 3.75 mg	2	0	–
Zopiclone 7.5 mg	1	3	–

All participants had to meet the following inclusion criteria: possession of a valid driving license for at least 3 years; average driving experience of at least 3,000 km per year over the last 3 years; mentally and physically fit to drive; good health based on a pre-study physical examination, medical history, vital signs, electrocardiogram, blood biochemistry, hematology, serology and urinalysis; body mass index between 19 and 30 kg/m^2^.

Exclusion criteria were history of drug or alcohol abuse; presence of a significant medical, neurological, psychiatric disorder, or sleep disorder other than insomnia; chronic use of medication that affects driving performance, except hypnotics; drinking more than 6 cups of coffee per day; drinking more than 21 alcohol containing beverages per week; and smoking more than 10 cigarettes per day.

Participants were screened for major psychopathology by use of the Symptom Checklist 90 Revised (Derogatis 1983), the Beck Depression Inventory (Beck et al. 1961), the State-Trait Anxiety Inventory (Spielberger et al. 1983), and the Multidimensional Fatigue Inventory (Smets et al. 1995).

During participation, use of caffeine was prohibited from 8 h prior to arrival on test days, until discharge the next morning. Alcohol intake was not allowed from 24 h prior to each dosing until discharge. Smoking was prohibited from 1 h prior to bedtime until discharge.

The study was conducted in accordance with the code of ethics on human experimentation established by the World Medical Association’s Declaration of Helsinki (1964) and amended in Edinburgh (2000). The protocol was approved by the medical ethics committee of Maastricht University and University Hospital of Maastricht. Subjects were explained the aims, methods, and potential hazards of the study and they signed a written informed consent prior to any study-related assessments.

### Design and treatments

The study was conducted according to a 3 × 2 double-blind, placebo controlled crossover design, with three groups (16 insomnia patients chronically using hypnotics, chronic users; 16 insomnia patients not or infrequently using hypnotics, infrequent users; and 16 self-defined good sleepers, matched for age and driving experience, controls) and two treatment conditions. Treatments were single oral doses of zopiclone 7.5 mg and placebo administered in identical looking capsules and ingested immediately before retiring to bed at 2330 hours. Treatments orders were balanced within groups (placebo–zopiclone or vice versa). Washout periods between treatments were at least 1 week.

In order to minimize withdrawal symptoms during the placebo night, patients assigned to the chronic users group were instructed to discontinue their hypnotic intake three nights before each treatment period. Chronic users who expected difficulties during the three hypnotic-free nights were provided escape medication, consisting of zolpidem at a maximum of one dose of 10 mg per night, to be used only in case of intolerable withdrawal effects. Zolpidem 10 mg was selected to limit variability in hypnotic drugs used and because it is known to be free from residual effects when taken at bedtime before 8 h of sleep (Vermeeren 2004).

A total of five patients from the chronic users group used zolpidem as escape medication in the 3-day period before a treatment night. Of those, three patients used escape medication in the night preceding the zopiclone condition, and four patients used escape medication in the night preceding the placebo condition.

### Assessments

#### Sleep

Sleep during treatment nights was evaluated objectively by polysomnography using montage including electroencephalogram, electrooculogram, and electromyogram. Sleep stages were visually assessed by qualified technicians according to standardized criteria (Iber et al. 2007). Sleep continuity parameters derived after analysis are sleep onset latency (in minute), wake after sleep onset (in minute), total sleep time (in minute), sleep efficiency (in percent), and number of awakenings. Sleep architecture parameters are percentages in stage 1, stage 2, slow wave, and REM of the total sleep time.

Upon arising, subjects completed the GSQS-spec (Mulder-Hajonides van der Meulen 1981). In addition, subjects estimated sleep onset latency (in minute), total sleep time (in minute), time awake before rising (in minute), and number of awakenings.

#### Driving performance

Driving performance was assessed using two on-the-road driving tests, a highway driving test, and a car following test. The Highway Driving Test (O’Hanlon 1984) measures road tracking performance that is mainly determined by the delay lag between sensory information, execution of motor reaction, and the vehicle’s dynamic response. In this test, subjects operate a specially instrumented vehicle over a 100-km (61-mi) primary highway circuit, accompanied by a licensed driving instructor having access to dual controls. The subjects’ task is to maintain a constant speed of 95 km/h (58 mi/h) and a steady lateral position between the delineated boundaries of the slower traffic lane. The vehicle speed and lateral position are continuously recorded. These signals are edited off line to remove data recorded during overtaking maneuvers or disturbances caused by roadway or traffic situations. The remaining data are then used to calculate means and standard deviations of lateral position and speed. Standard deviation of lateral position (SDLP in centimeters) is the primary outcome variable. SDLP is a measure of road tracking error or “weaving.” The test duration is approximately 1 h.

The Car-Following Test measures changes in controlled information processing such as selective attention, stimulus interpretation and decision making, and speed of an adaptive motor response to events which are common in driving (Brookhuis et al. 1994; Ramaekers and O’Hanlon 1994). In the test, two vehicles travel in tandem over a two-lane, undivided, secondary highway at 70 km/h (44 mi/h). An investigator drives the leading car and the subject, in the second car, is instructed to follow at a distance between 25 and 35 m. Subjects are further instructed to constantly attend the leading car since it may slow down or speed up at unpredictable times. They are required to follow the leading car’s speed movements, i.e., maintain the initial headway by matching the velocity of the car to the other’s. During the test, the speed of the leading car is automatically controlled by a modified “cruise control” system. At the beginning, it is set to maintain a constant speed of 70 km/h and, by activating a microprocessor, the investigator can start sinusoidal speed changes reaching amplitude of −10 km/h and returning to the starting level within 50 s. The maneuver is repeated six times. The leading car’s speed and signals indicating the beginning of the maneuver are transmitted via telemetry to be recorded in the following vehicle together with the following vehicle’s speed. Phase-delay converted to a measure of the subject’s average reaction time to the movement of the leading vehicle (RT, in second) is taken as the primary dependent variable in this test. A secondary measure in the Car Following Test is Gain. It represents an amplification factor between the signals of the two cars. This will be larger than 1 when the subject overreacts to speed adaptations of the leading car. Test duration is approximately 25 min.

#### Cognitive and psychomotor performance

Cognitive and psychomotor performance was assessed by use of a battery of laboratory tests for word learning, digit span, critical tracking, divided attention, psychomotor vigilance, and inhibitory control. Tests were previously proven to be sensitive to daytime sleepiness or sedation due to use of hypnotics (Vermeeren et al. 1995, 1998, 2002b; Verster et al. 2002; Leufkens et al. 2009; Leufkens and Vermeeren 2009).

The Word Learning Test, based on the Rey Auditory Verbal Learning Test (Rey 1964), is a verbal memory test for the assessment of immediate recall, delayed recall, and recognition performance. Fifteen monosyllabic nouns are presented and at the end of the sequence the subject is asked to recall as many words as possible. This procedure is repeated five times and after a delay of at least 30 min the subject is again required to recall as many words as possible. At this trial, the nouns are not presented. Finally, a sequence of 30 monosyllabic nouns is presented, containing 15 nouns from the original set and 15 new nouns in random order. The subject has to indicate whether a noun originates from the old set or it is from a new set of nouns.

The Psychomotor Vigilance Task (PVT, Dinges and Powell 1985) is based on a simple visual RT test. Subjects are required to respond to a visual stimulus presented at variable interval (2,000 to 10,000 ms) by pressing either the right or the left button with the dominant hand. The visual stimulus is a counter turning on and incrementing from 0 to 60 s at 1-ms intervals. In response to the subject’s button press, the counter display stops incrementing, allowing the subject 1 s to read the RT before the counter restarts. If a response has not been made in 60 s, the clock resets and the counter restarts. The test duration is 10 min. The average reaction time and the number of lapses (i.e., response times >500 ms) were used as the main performance parameters.

The Critical Tracking Test measures the ability to control an unstable signal in a tracking task. The signal deviates horizontally from a midpoint and the subject has to compensate this signal deviation by moving a joystick in opposite direction The test includes five trials of which the lowest and the highest score are discarded (Jex et al. 1966).

The Divided Attention Task measures the ability to divide attention between two simultaneously performed tasks (Moskowitz 1973). The first task is to perform a tracking test at a fixed level of difficulty set at 50 % of the maximum score obtained after extensive training of the Critical Tracking Test. In the other task, the subject has to monitor 24 single digits that are presented in the four corners of the screen. The digits change asynchronously at 5-s intervals. The subjects are instructed to remove their foot from a pedal as rapidly as possible whenever the digit “2” appears. This signal occurs twice at every location, in random order, at intervals of 5 to 25 s. Task duration is fixed at 12 min. The main performance parameters are average tracking error (in millimeter) and speed of target detection in the visual search task (in millisecond).

In the Stop Signal Task, the concept of inhibitory control is defined as the ability to stop a pending thought or action and to begin another (Logan et al. 1984). The paradigm consists of two concurrent tasks, i.e., a go task (primary task) and a stop task (secondary task). The go signals (primary task stimuli) are two letters (“X” or “O”) presented one at a time in the center of a computer screen. Subjects are required to respond to each letter as quickly as possible by pressing one of two response buttons. Occasionally, a stop signal (secondary task stimulus) occurs during the test. The stop signal consists of an auditory cue, i.e., a 1,000-Hz tone that is presented for 100 ms. The interval at which the stop signal is presented is dependent on the subject’s own successful and unsuccessful inhibitions. Subjects are required to withhold any response when a stop signal is presented. By continuously monitoring the subject’s response, the stop signal reaction time is calculated during the task. Subjects are required to withhold any response when a stop signal is presented.

#### Subjective evaluations

Subjective evaluations of driving quality, sedation, and mood were assessed using a series of visual analogue scales (100 mm). The driving instructors rated each subject’s driving quality and apparent sedation at the conclusion of the Highway Driving Test, using two 100-mm visual analogue scales. Subjects rated the extent of influence of the drug on their driving performance, prior to and upon completion of the Highway Driving Test, using a 100-mm visual analogue scale. In addition, subjects rated the degree of effort they had to put in driving performance using the Rating Scale Mental Effort (Zijlstra 1993). The scale is a visual analogue scale (150 mm) with additional verbal labels.

The subjects were instructed to rate their subjective feelings of alertness and sleepiness before the start of cognitive testing using the Karolinska Sleepiness Scale with scores ranging from 1 (extremely alert) to 9 (very sleepy, fighting sleep) (Akerstedt and Gillberg 1990) and a 16-item mood scale which provides three-factor analytically defined summary scores for “alertness,” “contentedness,” and “calmness” (Bond and Lader 1974).

#### Blood samples

Blood samples were taken at 9 1/2 h after ingestion of zopiclone 7.5 mg to determine serum concentrations of hypnotics before driving. Samples were centrifuged after a clotting period, and serum was frozen at −20 °C until analyses for pharmacokinetic assessments.

### Procedure

Subjects of all three groups were individually trained to perform the laboratory tests during two sessions of approximately 1.5 h in a previous study (Leufkens et al. 2014; Ramaekers 2011). In that study, they underwent two nights of sleep evaluation at the research facilities of the university. Subjects were therefore sufficiently familiarized with the testing facilities and procedures.

Treatment periods started in the evening of day 1, when the subjects arrived at the site at approximately 2000 hours, and lasted until day 2, when they were transported home after the driving test, at approximately 1145 hours. On arrival at the sleeping facility in each treatment period, subjects’ eligibility was verified. They were questioned about adverse events and use of medication since their last visit. Hereafter, electrodes for polysomnographic recording were attached.

Subjects ingested their medication and retired to bed at 2330 hours. They were awakened at 0730 hours and served a light standardized breakfast. At 0800 hours (i.e., 8.5 h post dose), they filled out the subjective rating scales for sleep, mood, and daytime sleepiness, and started the laboratory tests. At approximately 0900 hours, a blood sample was taken. Subjects were subsequently transported to the start of the highway driving test. Before driving, they rated the anticipated effect of the drug on their driving performance and performed the highway driving test between 0930 and 1030 hours (i.e., 1000–1100 hours post-dose). Upon completion, subjects were asked to rate the mental effort it took to perform the driving test and to evaluate the influence of the drug on their driving performance. Next, subjects performed the car following test, after which they returned to the testing facilities for removal of the electrodes.

### Statistical analysis

Sample size was based on a power calculation for detecting a clinically relevant effect of 2.4 cm in the primary measure of this study, the SDLP. This change corresponds to the effects of alcohol on SDLP, while blood alcohol concentrations (BACs) are 0.5 g/L as measured in a previous study (Louwerens et al. 1987). Given a test–retest reliability of SDLP of at least *r* = 0.70, a group of 16 subjects should permit detection of a mean change in SDLP of 2.0 cm, with a power of at least 90 % and an α risk of 0.05.

Overall effects were analyzed using a mixed model analysis of variance with *Group* as between subject factor with three levels (chronic users, infrequent users, controls) and *Treatment* as within subjects factor with two levels (zopiclone, placebo). Significant (*p* < 0.05) main effects or interactions were further analyzed using three univariate comparisons between groups for each treatment, and paired *t* tests between placebo and zopiclone within each group. Finally, zopiclone–placebo changes in SDLP were correlated with serum levels of zopiclone for each group separately.

All statistical analyses were done by using the Statistical Package for the Social Sciences (SPSS) statistical program (version 15.0 for Windows; SPSS, Chicago, IL).

## Results

### Driving performance

Out of 96 driving tests, one was terminated before scheduled completion because the driving instructor judged that it would be unsafe to continue. The subject was a female insomnia patient from the infrequent users group who had been administered zopiclone. Her SDLP score was calculated from the data collected until termination of the ride.

**Fig. 1:**
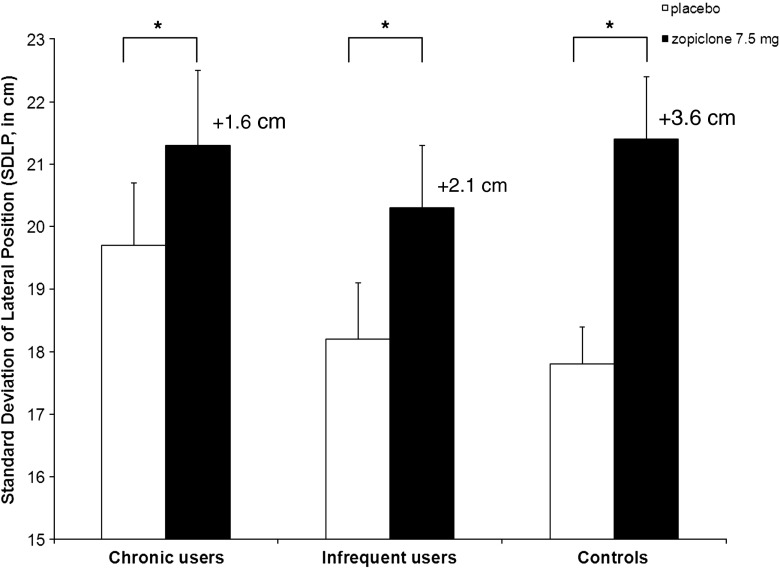
Mean (±SE) SDLP for each group separately (*p < 0.05, significant drug effect)

Figure 1 presents mean ± SE SDLP values recorded after placebo and zopiclone 7.5 mg for each group separately.

Analysis showed a highly significant overall treatment effect on SDLP (*F*_1,45_ = 33.86, *p* < 0.001). Zopiclone significantly impaired driving in all groups. Compared to placebo, the increase in SDLP was +1.6 cm (*p* = 0.010) in the chronic users group, +2.1 cm (*p* = 0.020) in the infrequent users group and +3.6 cm (*p* < 0.001) in the healthy control group. *t* tests for independent samples showed that the mean increase in SDLP from placebo to zopiclone was significantly lower in the chronic users group than the control group (*p* = 0.045). There was no difference between the infrequent users and controls and between the two insomnia groups.

Standard Deviation of Speed (Table 1) showed a significant main effect of treatment (*F*_1,45_ = 12.24, *p* = 0.001) and a treatment by group interaction (*F*_2,45_ = 3.42, *p* = 0.041).

Overall, zopiclone significantly impaired subjects’ control over speed variability. Paired *t* tests showed that zopiclone significantly increased SDSP in the control group (*p* < 0.004) and the chronic users group (*p* = 0.009), but not in the infrequent users group. There were no significant overall group effects on SDSP.

There were no overall effects on any of the Car Following Test parameters.

### Subjective evaluations of driving performance

Mean ± SE scores of the subjective evaluations of driving performance are presented in Table 2. The driving instructors did not judge the subjects’ driving quality and appearance of being sedated to be significantly different between zopiclone and placebo in all groups.

**Table 2:** Mean (±SE) scores of driving tests and subjective evaluations for each condition and group separately

Variable	Group				Statistics (*p* values)		
	Treatment	Chronic users	Infrequent users	Controls	Treatment × group	Treatment	Group
Highway Driving Test							
SDLP (cm)	Placebo	19.7 (1.0)	18.2 (0.9)	17.8 (0.6)	NS	<0.001	NS
	Zopiclone	21.3 (1.2)^a^	20.3 (1.0)^a^	21.4 (1.0)^a^			
SDSP (km/h)	Placebo	2.2 (0.1)	2.3 (0.2)	2.1 (0.1)	0.041	0.001	NS
	Zopiclone	2.5 (0.2)^a^	2.4 (0.1)	2.5 (0.4)^a^			
Car following test							
Reaction time (s)	Placebo	4.3 (0.5)	4.7 (0.4)	4.2 (0.6)	NS	NS	NS
	Zopiclone	4.5 (0.3)	3.8 (0.5)	4.4 (0.6)			
Gain	Placebo	1.2 (0.05)	1.1 (0.03)	1.2 (0.06)	NS	NS	NS
	Zopiclone	1.2 (0.06)	1.2 (0.07)	1.1 (0.03)			
Subjective evaluations by driving instructors							
Driving quality	Placebo	58.4 (5.8)	58.8 (4.6)	59.2 (5.0)	NS	NS	NS
	Zopiclone	58.6 (4.7)	61.1 (4.5)	55.8 (3.8)			
Apparent sedation	Placebo	18.9 (5.4)	15.1 (5.0)	18.4 (6.0)	NS	NS	NS
	Zopiclone	23.4 (4.3)	21.8 (3.2)	21.8 (4.4)			
Subjective evaluations by participants							
Anticipated driving quality	Placebo	71.7 (5.6)	73.6 (4.8)	86.2 (3.5)	NS	0.017	NS
	Zopiclone	67.6 (6.2)	71.7 (5.9)	66.4 (5.5)^a^			
Experienced driving quality	Placebo	67.9 (4.9)	65.0 (4.5)	78.3 (4.3)	NS	0.020	NS
	Zopiclone	60.8 (5.4)	62.4 (6.1)	66.8 (5.8)^a^			
Mental effort	Placebo	31.4 (5.3)	35.4 (5.4)	24.8 (4.7)	NS	<0.001	NS
	Zopiclone	45.1 (8.9)	53.3 (7.9)^a^	45.1 (6.8)^a^			
Karolinska Sleepiness Scale	Placebo	5.1 (0.4)^b^	4.4 (0.5)	3.5 (0.3)	NS	NS	0.009
	Zopiclone	5.1 (0.4)^b^	4.6 (0.4)	3.7 (0.3)			
Alertness	Placebo	58.8 (4.5)^b^	64.9 (4.1)	73.9 (3.4)	0.013	NS	NS
	Zopiclone	69.2 (3.9)^a^	65.8 (2.8)	66.2 (4.7)^a^			
Contentedness	Placebo	68.3 (3.8)	72.0 (3.4)	76.5 (4.2)	0.048	NS	NS
	Zopiclone	75.3 (3.5)	73.7 (3.7)	71.3 (4.5)			
Calmness	Placebo	72.6 (3.9)	75.9 (3.9)	75.2 (3.7)	NS	NS	NS
	Zopiclone	71.2 (3.5)	74.1 (4.0)	76.0 (3.6)			

*NS* no significant effect

^a^Significantly different from placebo within group (*p* < 0.05)

^b^Significantly different from control group (*p* < 0.05)

Overall, subjects’ ratings of anticipated and experienced driving quality were lower after zopiclone as compared to placebo (anticipated: *F*_1,44_ = 6.14, *p* = 0.017; experienced: *F*_1,44_ = 5.79, *p* = 0.020). Paired *t* tests showed that these differences reached significance within the control group, but not in the patient groups. Healthy controls expected driving quality to be worse after zopiclone administration (*p* = 0.006) and they confirmed this expectation after the driving test (*p* = 0.013). Changes in the patient groups were in the same direction, but smaller. *t* tests for independent samples revealed that both insomnia groups rated their driving quality in the placebo condition significantly lower than the control group (chronic users: *p* = 0.040; infrequent users: *p* = 0.045).

Overall, subjects’ perceived mental effort to perform the driving test was increased after zopiclone as compared to placebo (*F*_1,45_ = 16.15, *p* < 0.001). Paired *t* tests showed that this difference reached significance within the control group (*p* = 0.009) and the infrequent users group (*p* = 0.008), but not in the chronic users group. Differences between groups were not significant.

### Subjective evaluations of sleepiness and feelings

Mean ± SE scores of the subjective evaluations of sleepiness and feelings are presented in Table 2. Subjects’ ratings of sleepiness as measured by the Karolinska Sleepiness Scale were significantly different between groups (*F*_2,44_ = 5.28, *p* = 0.009), but not between treatments. The chronic users group felt more sleepy than the healthy controls after placebo (*p* = 0.003) and after zopiclone (*p* = 0.023) administration.

Subjective feelings of alertness and contentedness showed a significant treatment by group interaction (*F*_2,45_ = 4.81, *p* = 0.013 and *F*_2,45_ = 3.25, *p* = 0.048, respectively). In the placebo condition, the insomnia groups felt significantly less alert than the control group. This difference was significant for the chronic users group (*p* = 0.011). Use of zopiclone increased next day alertness in the chronic users (*p* = 0.029), whereas it impaired alertness in the healthy controls (*p* = 0.040). For feelings of contentedness, further analyses did not reveal differences between treatments and groups.

There were no overall main effects for subjective feelings of calmness.

### Cognitive and psychomotor assessment

Table 3 summarizes the mean ± SE scores of the cognitive performance tests.

**Table 3:** Mean (±SE) scores of cognitive performance tests for each condition and group separately

Variable	Group				Statistics (*p* values)		
	Treatment	Chronic users	Infrequent users	Controls	Treatment × group	Treatment	Group
Word Learning Test							
Immediate recall score	Placebo	39.5 (1.9)	43.6 (2.2)	46.2 (1.9)	NS	0.003	NS
	Zopiclone	35.4 (2.9)	39.1 (2.7)^a^	41.6 (2.5)			
Delayed recall	Placebo	5.3 (0.7)	6.8 (0.9)	7.3 (0.8)	NS	0.024	NS
	Zopiclone	4.8 (0.8)	5.8 (0.8)	5.4 (0.7)			
Recognition score	Placebo	25.4 (1.1)	25.0 (1.3)	25.5 (1.3)	NS	0.014	NS
	Zopiclone	24.4 (0.8)	23.4 (1.4)	23.5 (1.5)^a^			
Recognition reaction time (ms)	Placebo	848 (32)	785 (36)	851 (33)	NS	0.006	NS
	Zopiclone	889 (39)	863 (40)	893 (34)			
Psychomotor Vigilance Task							
Average reaction time (ms)	Placebo	290 (13)	290 (15)	281 (8)	NS	NS	NS
	Zopiclone	288 (12)	303 (16)	297 (11)			
Lapses (>500 ms)	Placebo	3.1 (1.0)	1.7 (0.4)	2.1 (0.6)	NS	NS	NS
	Zopiclone	2.6 (0.8)	3.1 (1.0)	2.6 (0.6)			
Critical Tracking Task							
Average lambda (rad/s)	Placebo	3.1 (0.2)	2.9 (0.2)	2.8 (0.2)	NS	0.007	NS
	Zopiclone	2.9 (0.2)	2.7 (0.2)	2.7 (0.1)			
Divided Attention Task							
Average error (mm)	Placebo	16.5 (1.5)	18.4 (1.1)	19.8 (1.3)	NS	<0.001	NS
	Zopiclone	18.9 (1.2)^a^	20.5 (1.3)^a^	21.4 (1.1)			
Reaction time (ms)	Placebo	2,052 (87)	1,971 (75)	1,941 (81)	NS	0.034	NS
	Zopiclone	2,038 (65)	2,086 (71)^a^	2,140 (93)			
Stop Signal Task							
Hits (#)	Placebo	241 (3)	246 (1)	247 (1)	NS	0.016	NS
	Zopiclone	240 (3)	245 (1)	245 (2)			
Go reaction time (ms)	Placebo	437 (19)	432 (19)	438 (13)	NS	0.001	NS
	Zopiclone	442 (18)	452 (20)^a^	459 (15)^a^			
Stop reaction time (ms)	Placebo	181 (7)	184 (8)	202 (10)	.034	<0.001	0.016
	Zopiclone	184 (9)^b^	197 (8)^a^	229 (9)^a^			

NS = no significant effect

^a^Significantly different from placebo within group (*p* < 0.05)

^b^Significantly different from control group (*p* < 0.05)

Overall, zopiclone impaired all parameters of the Word Learning Test, i.e., immediate recall (*F*_1,45_ = 9.78, *p* = 0.003), delayed recall (*F*_1,45_ = 5.49, *p* = 0.024), recognition score (*F*_1,45_ = 6.48, *p* = 0.014), and recognition reaction time (*F*_1,45_ = 8.50, *p* = 0.006). Paired *t* tests revealed that these effects did not reach significance in each group separately, except for the effect on immediate recall in the infrequent users group (*p* = 0.039), and the effects on the recognition score in the control group (*p* = 0.045). *t* tests for independent samples revealed that the chronic users scored significantly worse than the healthy controls on the immediate recall score in the placebo condition (*p* = 0.020). Other significant group differences were not found.

Performance in the Psychomotor Vigilance Task did not show significant overall differences between treatments and groups.

Overall, zopiclone affected psychomotor performance in the Critical Tracking Task (*F*_1,44_ = 8.15, *p* = 0.007). Analysis for groups separately did not reveal significant treatment effects, however.

In the Divided Attention Task, there was an overall significant impairment by zopiclone in both the tracking subtask (*F*_1,44_ = 16.49, *p* < 0.001) and the detection subtask (*F*_1,44_ = 4.81, *p* = 0.034). Tracking, as reflected by the average error, was significantly worse after zopiclone administration in both insomnia groups (chronic users: *p* = 0.024; infrequent users: *p* = 0.008), but not in the control group. Detection, as reflected by reaction time, was significantly impaired following zopiclone in the infrequent users group only (*p* = 0.048).

A significant overall treatment effect was found on the number of hits (*F*_1,43_ = 6.31, *p* = 0.016) and the go reaction time (*F*_1,43_ = 12.63, *p* < 0.001) in the Stop Signal Task. For the number of hits, paired *t* tests revealed that this effect did not reach significance in each group separately, however. Paired *t* tests did show that the go reaction time was significantly slower after zopiclone administration in the infrequent users (*p* = 0.026) and the controls (*p* = 0.033). Stop reaction time showed a significant main effect of treatment (*F*_1,43_ = 15.84, *p* = 0.001), a significant overall group difference (*F*_2,43_ = 4.58, *p* = 0.016) and a treatment by group interaction (*F*_2,43_ = 3.67, *p* = 0.034).

Overall, zopiclone significantly slowed subjects’ response to a stop signal. Paired *t* tests showed that zopiclone significantly increased stop reaction time in the infrequent users group (*p* = 0.046) and the control group (*p* = 0.007), but not in the chronic users group.

Overall analysis for group differences revealed a significant effect following zopiclone administration (*F*_2,44_ = 5.27, *p* = 0.009), but not following placebo administration. Post hoc analysis showed that the chronic users had significantly less problems with responding to a stop signal after zopiclone than the controls had (*p* = 0.002).

### Sleep quality

#### Subjective evaluation

Table 4 summarizes the mean ± SE scores of both the subjective and objective sleep parameters for each group separately after administration of placebo and zopiclone.

**Table 4:** Mean (±SE) scores of subjective and objective sleep quality

Variable	Group				Statistics (*p* values)		
	Treatment	Chronic users	Infrequent users	Controls	Treatment × group	Treatment	Group
Subjective							
Groningen Sleep Quality Scale	Placebo	10.8 (0.8)^a^	8.9 (0.9)^a^	4.2 (1.0)	0.022	<0.001	<0.001
	Zopiclone	5.3 (1.0)^a, b^	3.3 (0.6)^b^	2.4 (0.5)			
Sleep onset time (min)	Placebo	114 (22)^a, c^	53 (11)	36 (8)	0.049	0.001	<0.001
	Zopiclone	44 (11)^b^	27 (4)	24 (4)			
Awakenings (#)	Placebo	3.5 (0.6)	3.8 (0.6)^a^	2.0 (0.4)	NS	<0.001	0.027
	Zopiclone	1.9 (0.4)^a, b^	1.4 (0.4)^b^	0.7 (0.2)^b^			
Total sleep time (min)	Placebo	241 (21)^a^	291 (23)^a^	385 (13)	0.012	<0.001	<0.001
	Zopiclone	355 (18)^a, b, c^	403 (12)^b^	419 (8)^b^			
Polysomnographic parameters							
Sleep onset time (min)	Placebo	42.8 (7.6)^c^	21.2 (4.3)	25.9 (4.5)	NS	0.031	0.008
	Zopiclone	31.3 (4.8)^c^	16.6 (2.6)	22.5 (2.6)			
Wake after sleep onset (min)	Placebo	94.1 (11.4)	73.5 (9.2)	73.6 (13.2)	NS	<0.001	0.029
	Zopiclone	78.2 (12.6)^a, c^	48.4 (5.2)^b^	35.9 (4.5)^b^			
Awakenings (#)	Placebo	8.7 (1.2)	7.6 (0.8)	7.3 (1.1)	NS	0.004	NS
	Zopiclone	7.5 (1.0)	5.9 (0.8)	4.4 (0.8)^b^			
Total sleep time (min)	Placebo	343 (15)	386 (11)	381 (13)	NS	<0.001	0.002
	Zopiclone	372 (14)^a, b, c^	414 (6)^b^	423 (5)^b^			
Sleep efficiency (%)	Placebo	71.5 (3.1)	80.4 (2.4)	79.6 (2.7)	NS	<0.001	0.002
	Zopiclone	77.3 (2.9)^a, b, c^	86.9 (1.0)^b^	88.6 (0.7)^b^			
Stage 1 sleep (% of total sleep time)	Placebo	7.9 (0.9)	6.7 (0.8)	7.2 (1.1)	NS	0.007	NS
	Zopiclone	7.5 (1.0)	4.8 (0.6)^b^	5.1 (0.8)^b^			
Stage 2 sleep (% of total sleep time)	Placebo	55.9 (2.1)	51.5 (1.6)	53.2 (2.3)	NS	0.009	NS
	Zopiclone	59.9 (2.2)	54.1 (2.0)	55.9 (2.2)			
Stage SWS sleep (% of total sleep time)	Placebo	15.1 (2.3)	20.6 (1.9)	20.7 (2.2)	NS	0.001	NS
	Zopiclone	18.5 (2.4)	24.4 (2.1)^b^	21.9 (2.2)			
Stage REM sleep (% of total sleep time)	Placebo	20.9 (1.2)	21.1 (1.3)	18.9 (1.0)	0.027	<0.001	NS
	Zopiclone	14.1 (0.8)^b^	16.8 (1.3)^b^	17.0 (1.0)			

*NS* no significant effect

^a^Significantly different from control group (*p* < 0.05)

^b^Significantly different from placebo within group (*p* < 0.05)

^c^Significantly different from infrequent users group (*p* < 0.05)

Significant overall main effects of treatment and group were found for all parameters (number of complaints: *F*_1,45_ = 49.34, *p* < 0.001; sleep onset time: *F*_1,45_ = 14.05, *p* = 0.001; number of awakenings: *F*_1,45_ = 33.56, *p* < 0.001; total sleep time: *F*_1,44_ = 52.29, *p* < 0.001). In addition, significant interactions showed that the effect of zopiclone differed between groups in number of complaints (*F*_2,45_ = 4.18, *p* = 0.022), sleep onset time (*F*_2,45_ = 3.24, *p* = 0.049), and total sleep time (*F*_2,44_ = 4.90, *p* = 0.012). Paired *t* tests of treatment effect for each group separately showed that in both insomnia groups number of sleep complaints was significantly reduced (both groups: *p* < 0.001) and total sleep time was significantly increased (chronic users: *p* < 0.001; infrequent users: *p* = 0.001) after zopiclone administration. Number of awakenings was significantly reduced after zopiclone in all groups (chronic users: *p* = 0.022; infrequent users: *p* < 0.001; controls: *p* = 0.008). Sleep onset time was significantly diminished after zopiclone in the chronic users group only (*p* = 0.014).

Significant differences between chronic users and controls were found in number of complaints (*p* < 0.001), sleep onset time (*p* = 0.006), and total sleep time (*p* < 0.001) after placebo. Following zopiclone administration, chronic users reported significantly more sleep complaints (*p* = 0.007), shorter total sleep time (*p* = 0.001), and more awakenings (*p* = 0.015) than controls.

Comparisons between the infrequent users and the controls revealed that there were significant differences in number of complaints (*p* = 0.001), total sleep time (*p* = 0.001), and number of awakenings (*p* = 0.027) after administration of placebo. Differences between the groups disappeared after zopiclone administration.

Comparisons between the insomnia groups showed that the chronic users had a significantly longer sleep onset time (*p* = 0.006) and a significantly shorter total sleep time (*p* = 0.001) than the infrequent users in the placebo condition. There were no differences between the insomnia groups after zopiclone administration.

#### Polysomnographic parameters

Significant overall main treatment effects were found in all polysomnographic parameters for sleep continuity (sleep onset time: *F*_1,42_ = 4.97, *p* = 0.031; wake after sleep onset: *F*_1,41_ = 18.71, *p* < 0.001; number of awakenings: *F*_1,42_ = 9.36, *p* = 0.004; total sleep time: *F*_1,42_ = 23.30, *p* < 0.001; sleep efficiency: *F*_1,41_ = 23.26, *p* < 0.001). Paired *t* tests showed that following zopiclone administration all groups slept significantly longer (chronic users: *p* = 0.033; infrequent users: *p* = 0.014; controls: *p* = 0.006) and improved their sleep efficiency (chronic users: *p* = 0.034; infrequent users: *p* = 0.012; controls: *p* = 0.009). Furthermore after zopiclone, the infrequent users and controls had significantly less time awake after sleep onset (*p* = 0.016 and *p* = 0.017, respectively). Lastly, the controls had significantly fewer awakenings after zopiclone administration as compared to placebo (*p* = 0.027).

Except in the number of awakenings, significant overall group effects were found in all objective evaluations (sleep onset time: *F*_2,42_ = 5.47, *p* = 0.008; wake after sleep onset: *F*_2,41_ = 3.86, *p* = 0.029; total sleep time: *F*_2,42_ = 7.11, *p* = 0.002; sleep efficiency: *F*_2,41_ = 7.61, *p* = 0.002). Following zopiclone administration and compared to both the controls and infrequent users, the chronic users had a significantly longer wake after sleep onset time (*p* = 0.001 and *p* = 0.033, respectively), significantly less total sleep time (*p* = 0.001 and *p* = 0.004, respectively), and significantly worse sleep efficiency (*p* < 0.001 and *p* = 0.001, respectively). Sleep onset time appeared to be significantly longer for the chronic users as compared to the infrequent users after both placebo (*p* = 0.029) and zopiclone administration (*p* = 0.010). No differences between infrequent users and controls were found on any of the parameters for sleep continuity after placebo or zopiclone administration.

Overall main effects of treatment were also found in all parameters for sleep architecture (percentage of stage 1 sleep: *F*_1,42_ = 8.13, *p* = 0.007; percentage of stage 2 sleep: *F*_1,42_ = 7.40, *p* = 0.009; percentage of slow wave sleep: *F*_1,42_ = 11.57, *p* = 0.001; percentage of REM sleep: *F*_1,42_ = 37.10, *p* < 0.001). In the infrequent users, stage 1 sleep and REM sleep were significantly reduced after zopiclone administration (*p* = 0.028 and *p* = 0.001, respectively) and slow wave sleep was significantly increased (*p* = 0.001). Zopiclone also significantly decreased the percentage of stage 1 sleep in the controls (*p* = 0.042) and REM sleep in the chronic users (*p* < 0.001).

Overall main group differences were not found on any of the parameters. A significant treatment by group interaction was shown in the percentage of REM sleep. Yet, further analysis did not reveal any group differences.

### Serum concentrations

Mean (±SE) serum concentrations for zopiclone were 9.9 (1.0) ng/mL in the chronic users group, 11.3 (1.2) ng/mL in the infrequent users group, and 10.7 (0.7) ng/mL in the controls group. Overall group analysis revealed no significant differences between the three groups.

Correlations between change in SDLP from placebo to zopiclone and serum concentrations were 0.06 (n.s.) for the chronic users, 0.48 (n.s.) for the infrequent users, and 0.54 (*p* < 0.05) for the controls.

## Discussion

Results of the present study show that a single oral dose of zopiclone 7.5 mg significantly impairs on-the-road driving performance in insomnia patients who chronically use hypnotics, in insomnia patients who not or infrequently use hypnotics, and in healthy, good sleepers at 10 to 11 h after bedtime administration. The impairing effect of zopiclone on driving, as reflected by the rise in SDLP compared with placebo, was significantly different between the chronic users and the healthy controls. As a result, interpretation of the severity of the effects differs between groups. The effect found in the chronic users group (+1.6 cm) is on average of lesser magnitude than that produced by alcohol in a previous study while subjects drove with BAC of 0.5 mg/mL (+2.4 cm) (Louwerens et al. 1987), which is the legal limit for driving a car in most countries. In contrast, the increase of 3.6 cm in SDLP from placebo after zopiclone 7.5 mg administration in the healthy control group is above this effect of alcohol. Zopiclone produced an effect of +2.1 cm in the infrequent users group, which was slightly less than that of alcohol while BACs are 0.5 mg/mL. However, there was no statistical difference in the effect of zopiclone between the infrequent users and the controls.

The significantly decreased magnitude of effect of zopiclone 7.5 mg on driving performance in the chronic users as compared to the healthy controls suggests that residual effects are attenuated by chronic use of hypnotics. This may be explained by the development of tolerance towards the impairing effects of zopiclone even for those patients using hypnotics other than zopiclone as cross-tolerance from benzodiazepines to zopiclone has been shown previously (Lader 1997). This implicates that results from studies conducted with healthy volunteers appear to give an overestimation of the actual effects in insomnia patients chronically using hypnotics. It should be mentioned, however, that SDLP scores following zopiclone administration were still significantly increased in the chronic users group, indicating that residual effects do not completely disappear.

An alternative explanation for the decreased effect of zopiclone on driving in chronic users may be the occurrence of withdrawal symptoms in the placebo condition. Mean SDLP scores after zopiclone were comparable between the three groups. After placebo, however, mean SDLP in the chronic users group was elevated compared with the infrequent users and healthy controls, although the difference did not reach statistical significance. This indicates worse performance and suggests that withdrawal symptoms may have been present in the chronic users despite discontinuation of their own hypnotic intake 3 days before each treatment period. The patients may have experienced discomfort from the hypnotic-free night which may have impaired their driving performance. So, the difference in SDLP scores between placebo and zopiclone in the chronic users may be relatively small due to their bad performance in the placebo condition. Yet, according to polysomnographic analyses, sleep in the chronic users appeared not to be significantly affected by possible withdrawal effects during the placebo night. Besides a significant difference of 20 min in sleep onset as compared to the infrequent users, the sleep profile of the chronic users was comparable with that of the other two groups. The only support for reduced ability to perform in the chronic users due to withdrawal symptoms in the placebo condition may be the decreased feelings of alertness and increased feelings of sleepiness as compared to the controls following placebo administration. Even more, the chronic users reported feeling significantly more alert after the zopiclone night, whereas the controls felt the exact opposite. This may suggest that the chronic users experienced more discomfort after the placebo night than after the zopiclone night and may have influenced their driving performance. After all, this was their fourth hypnotic-free night in succession possibly causing physical problems (Pétursson 1994).

The magnitude of residual effects of zopiclone 7.5 mg found in the infrequent users group is in line with previous studies conducted in healthy younger drivers (Vermeeren et al. 1998, 2002b; Leufkens et al. 2009). In those studies, the mean increase in SDLP from placebo after zopiclone administration ranged from +2.5 to +4.9 cm. This suggests that the residual effects of hypnotics found in healthy volunteers can validly predict the effects in older patients suffering from insomnia.

The impairing effects on driving after zopiclone appeared not to be noticed by the insomnia patients. Prior to the start of the driving test, they did not expect to drive differently after administration of zopiclone than after placebo. In addition, after completion of the test, they did not rate their driving quality differently between zopiclone and placebo. In contrast, the healthy controls anticipated their driving quality to be significantly worse after zopiclone. Their expectations were confirmed by their performance as they evaluated their driving quality after the test significantly lower after zopiclone administration as compared with placebo.

Whereas the residual adverse effects of zopiclone remained undetected by the insomnia patients according to their subjective evaluations, opposite results were found for the evaluations of its therapeutic effects. Both insomnia groups reported significantly improved subjective sleep quality on most parameters after zopiclone administration as compared with placebo. The healthy controls, however, appeared to benefit considerably less from the sleep-inducing properties of zopiclone. According the objective measures of sleep, there were virtually no differences in sleep quality between the groups, however. Still, the chronic users group felt significantly more alert the morning after an evening dose of zopiclone than after placebo. The infrequent users did not report a difference in alertness, whereas the healthy controls reported feeling significantly less alert after zopiclone than after placebo.

The lack of awareness of residual sedative effects of zopiclone 7.5 mg may cause insomnia patients to belief that car driving is safe the morning after evening administration. Even more, these beliefs may be strengthened by the experienced improvement of subjective sleep quality. These results stress, however, the importance of general physicians to warn their patients about the impairing effects of zopiclone 7.5 mg on driving performance.

In contrast to the significant effects of zopiclone on the highway driving test, no impairment was found on the car following test. It appears that monotonous tasks, such as the highway driving test, are more sensitive to hypnotic-induced sedation than more complex, rule-based tasks, such as the car following test. A similar discrepancy of results has been found in a study investigating the effects of sleep deprivation on driving (Bosker et al. 2012). It is explained in that study that the car following test consists of stimuli generating interest and effort and, consequently, participants are able to compensate for their sleepiness. It may well be the case that a similar mechanism was present in the participants of the current study.

The impairing effects of zopiclone on highway driving performance could not be completely corroborated by the results of the cognitive performance tests. Although there were overall significant differences between placebo and zopiclone on a majority of the parameters, in about only half of the cases impairing effects of zopiclone were found in one or two specific groups. For instance, zopiclone only impaired immediate recall in the infrequent users group and recognition performance in the healthy controls. Evident impairing residual effects of zopiclone 7.5 mg on verbal learning have been found recently, however, in both healthy older and younger subjects (Leufkens et al. 2009; Leufkens and Vermeeren 2009). Average scores of the three groups in the present study following placebo administration appeared to be slightly lower already than the average scores of healthy older subjects in the previous study (Leufkens and Vermeeren 2009). This may suggest that scores were close to a minimum, showing possible floor effects.

Performance on the psychomotor vigilance task was not different between placebo and zopiclone administration. Although there is ample evidence that the PVT is highly sensitive to the effects of sleep deprivation (Lim and Dinges 2008), there seem to be almost no studies assessing effects of sedating drugs on this test. To our knowledge, the sedative residual effects of zopiclone 7.5 mg have not yet been investigated with use of this task. There are two recent studies showing significant effects of blood alcohol concentrations of 0.03 and 0.05 mg/mL on PVT performance in healthy subjects (Howard et al. 2007; Jongen et al. 2014). This suggests that the task should have been sufficiently sensitive to detect the residual effects zopiclone, which were comparable in magnitude to those of blood alcohol concentrations of 0.05 mg/mL as measured by SDLP. The failure to find an effect on the PVT therefore indicates that performance on this test is less sensitive to residual effects of GABAergic hypnotic drugs than acute effects of alcohol.

There are also limitations to this study. Sleep was not measured during the period that the chronic users were requested to refrain from taken their own hypnotics. A possible accumulation of sleep loss due to rebound insomnia caused by withdrawal from hypnotic use cannot be verified therefore. An indirect indication that withdrawal symptoms may have been mild is the number of chronic users taking zolpidem 10 mg as escape medication in the 3-day period before a treatment condition. Only five participants needed escape medication, but not on all days and 11 participants did not take any medication. Still, as has been reviewed extensively, rebound insomnia is very likely to occur after discontinuation of chronic use of hypnotics (Gillin et al. 1989; Kales et al. 1990).

Another limitation of this study may be that the effects on driving have only been investigated after a single dose of zopiclone. A study in which administration of a hypnotic occurred, for example, for at least a week could have better been able to show the effects of the development of tolerance. It may be expected in that case that the difference in effect between day 1 of dosing and day 8 of dosing would be smaller in chronic users than in infrequent users and healthy controls. A placebo condition of 1 week, however, is likely to be too long for chronic users creating a practical problem for such studies.

To summarize, results of the present study indicate that driving performance is mildly impaired in insomnia patients after evening administration of zopiclone 7.5 mg at least until 11 h after intake. Chronic use of hypnotics seems to attenuate the severity of effects of zopiclone 7.5 mg. Nevertheless, this reduction does not result in an absence of impairing effects in insomnia patients chronically using hypnotics. The magnitude of effects found in the infrequent users group was slightly smaller than found in previous studies investigating residual effects of zopiclone 7.5 mg in healthy, younger volunteers. This suggests that investigating residual effects of hypnotics in healthy volunteers may yield a minor overestimation of the actual effects in insomnia patients who start using hypnotics.
